# Prevalence and risk factors of fractures in transfusion dependent thalassemia – A Hong Kong Chinese population cohort

**DOI:** 10.1002/edm2.340

**Published:** 2022-04-30

**Authors:** Samantha Lai Ka Lee, Raymond Siu Ming Wong, Chi Kong Li, Wing Kwan Leung

**Affiliations:** ^1^ Division of Endocrinology Department of Paediatrics Hong Kong Children's Hospital Hong Kong SAR China; ^2^ Department of Paediatrics The Chinese University of Hong Kong Hong Kong SAR China; ^3^ Division of Haematology Department of Medicine Prince of UK Hospital Hong Kong SAR China; ^4^ Department of Medicine The Chinese University of Hong Kong Hong Kong SAR China; ^5^ Division of Haematology Department of Paediatrics Hong Kong Children 's Hospital Hong Kong SAR China

**Keywords:** bisphosphonates, bone mineral density, fracture, osteoporosis, thalassemia

## Abstract

**Objective:**

To delineate the prevalence and associated risk factors of low BMD, osteoporosis/bone fragility and fracture in transfusion‐dependent thalassemia (TDT) in the Chinese population in Hong Kong.

**Design, Patients and Measurements:**

A retrospective cohort study design was employed. Patients of TDT who had serial Hologic dual‐energy X‐ray absorptiometry (DXA) from 2010 to 2016 and received regular transfusion for at least 5 years were recruited. Clinical and biochemical data, from 5 years before the first DXA scan, were retrieved from the electronic record system of the Hospital Authority, till 30 June 2020. Low bone mineral density and osteoporosis/bone fragility are defined by the ISCD 2019 position guidelines.

**Results:**

Seventy‐seven patients were included in the analysis. The fracture prevalence of TDT among the Chinese population in Hong Kong was 15.58%. Up to 55.84% of patients had low bone mineral density, and 5.19% patients had osteoporosis/bone fragility state. The median age at first fracture was 31.73 years (range 24.06–44.18 years). In the regression analysis, a higher log(10) transformation of average ferritin levels over 5 years before the first DXA scan was significantly associated with fracture occurrence regardless of bisphosphonate treatment (OR 310.73, 95% CI 3.99–24183.89, *p* = .010). Mean average ferritin level over 5 years was 6695.5 ± 2365.7 pmol/L (fracture group) versus 4350.7 ± 3103.2 pmol/L (non‐fracture group), *p* = .016. Hip and spine BMD *Z*‐score did not have statistically significant association with fracture occurrence.

**Conclusion:**

Iron overloading plays an important role in adverse bone health in TDT. Dual X‐ray densitometry is insufficient in predicting fracture risk.

## Graphical abstract

The cohort of this study included participants with long follow‐up period and a large sample size to assess the fracture outcomes in a succinct way. The study has demonstrated average ferritin levels over a period of 5 years was significantly associated with fracture occurrence, suggesting for an insidious process of adverse impact of iron overloading in bone health. Our data has updated the prevalence of low mineral density, osteoporosis/ bone fragility state according to ISCD 2019 position guidelines, which allowed for avoidance of over‐labelling of osteoporosis with T‐score alone. Our data has suggested that dual X‐ray densitometry is insufficient in predicting fracture risk, though data was limited by the heterogeneous timing of the bone densitometry.

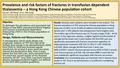

## 1 INTRODUCTION

Thalassemia is a common haemoglobinopathy in Southeast Asia. Around 5.2% of the world population is being affected by clinically significant β‐thalassemia or α^0^‐thalassaemia.^1^ Prior report showed 381 patients required regular transfusion in Hong Kong.^2^ Survival in patients with transfusion dependent thalassemia (TDT) improved with optimized care and newer iron chelating agents, long‐term complications surfaced as a result.^3^ Bone health has become a major issue in the past decades as this specific cohort is aging.^4^ The relationship between bone health and iron homeostasis is highly intricated.^5^ The exact mechanisms of bone disorder in thalassemia have yet to be fully understood.^6^ Commonly employed iron chelators for example, desferrioxamine (DFO), has been implicated in long bone dysplasia in thalassaemic patients.^7^ Moreover, hypoparathyroidism, diabetes mellitus or gonadal failure, as a result of iron overloading, pose additional adverse impact on bone health.^8^, ^9^ This study aims to describe the magnitude of osteoporosis/bone fragility based on the latest 2019 International Society for Clinical Densitometry (ISCD) position guideline^10^, ^11^ and to explore the prevalence and risk factors leading to fractures, a clinical oriented outcome, in patients of TDT.

## 2 MATERIALS AND METHODS

Patients of TDT underwent serial dual‐energy X‐ray absorptiometry (DXA) from 2010 to 2016 at Jockey Club Centre for Osteoporosis Care and Control of The Chinese University of Hong Kong as part of clinical surveillance for potential complication of iron overloading, which was participated on a voluntary basis. Patients who were on regular transfusion for at least 5 years prior to first DXA scan were invited to participate in the study. Informed consents were obtained for retrospective review of participants' clinical records. The clinical, biochemical and magnetic resonance imaging (MRI) T2* data, starting from 5 years prior to the first DXA scan until 30 June 2020, were retrieved from the ‘Electronic Patient Record System’ of the Hospital Authority for analysis. This study was approved by the ethics committee of the Hospital Authority of Hong Kong. The Hologic system was employed for DXA assessment. Definitions of low bone mineral density, bone fragility state and osteoporosis were based on the ISCD 2019 position guidelines (ISCD). Low bone mineral density was defined as height adjusted BMD *Z*‐score of less than or equal to −2.0 for the paediatric population, BMD *Z*‐score of less than or equal to −2.0 for premenopausal women or men <50 years, or BMD *T*‐score above −2.5 and below −1.0 in post‐menopausal women or men older than 50 years. Bone fragility was defined as presence of vertebral fracture or height‐adjusted lumbar spine BMD *Z*‐score less than or equal to −2.0 with clinically significant fractures in the paediatric population, premenopausal women, or men <50 year. BMD *T*‐score <−2.5 at lumbar spine, hip or femoral neck defined osteoporosis in post‐menopausal women or men older than 50 years.

## 3 RESULTS

A total of 77 patients (38 females and 39 males) were included in the analysis. Baseline characteristics were summarized in Table 1. The median age at first DXA was 29.28 years (range 10.67–67.18 years). The median duration of follow‐up was 9.18 years (range 5.35–9.53 years) from first DXA. In our cohort, 36 (46.75%), 11 (14.29%) and 2 (2.60%) patients had undergone two, three and four serial DXA scans respectively. The remaining 28 patients had undergone one DXA scan only. The median duration between first and second DXA scan was 1.72 years (range 0.86–5.16 years). The median duration between first and last DXA scans was 2.26 years (range 0.86–5.16 years). The mean ± SD of spinal *Z*‐score, total hip *Z*‐score, spinal *T*‐score and total hip *T*‐score at first DXA for the entire cohort were −1.79 ± 1.01 (*n* = 77), −1.70 ± 1.14 (*n* = 77), −2.14 ± 0.90 (*n* = 64) and − 1.97 ± 0.94 (*n* = 64) respectively. Forty‐three of 77 participants (55.84%) had low BMD (based on either spinal or total hip *Z*‐score −2.00 or lower). ISCD defined bone fragility/osteoporosis was 5.19%.

**TABLE 1:** Baseline clinical characteristics

Diagnosis	
Beta‐thalassemia	74 (96.10%)
Alpha‐thalassemia	3 (3.90%)
Transfusion‐related parameters	
Mean serum ferritin over 5 years prior DXA	4720.69 ± 3080.00 pmoL/L
Liver iron overload by MRI T2* (*n* = 70)	46 (mild 27, moderate 13, severe 6)
Cardiac iron overload by MRI T2* (*n* = 70)	13
Transfusion volume (ml/kg/year)	242.00 ± 50.02 ml/kg/year
Desferrioxamine (DFO) use	51 (63.5%), dose 1906.86 ± 497.40 mg/day
Deferiprone (L1) use	54 (70.13%), dose 3531.82 ± 1099.30 mg/day
Deferasirox use	13 (16.88%), dose 1276.92 ± 527.91 mg/day
Combination of DFO and oral chelators	41 (53.25%)
Anthropometric data at 1st DXA scan	
Mean height ± SD (cm)	154.48 ± 8.86
Mean weight ± SD (kg)	49.70 ± 8.89
Mean BMI ± SD (kg/m^2^)	20.76 ± 3.03
Calcium supplementation before 1st DXA scan	40 (51.95%)
Vitamin D_3_ supplementation before 1st DXA scan	32 (41.56%)
Gonadal dysfunction	39 (50.65%) – 33 on HRT
Hypoparathyroidism	5 (6.49%)
Hypothyroidism	26 (26.34%) – 22 had central hypothyroidism
Obesity	5 (6.49%)
DM	13 (16.88%) – T1DM 6, T2DM 7
Cardiomyopathy	11 (14.29%)

Abbreviations: DM, diabetes mellitus; DXA, dual‐energy bone densitometry; HRT, hormonal replacement therapy; MRI, magnetic resonance imaging; SD, standard deviation; T1DM, type 1 DM; T2DM, type 2 DM.

Fractures were documented in 12 individuals (15.58%) in the entire cohort, of whom one had recurrent fractures at femur. Four had sustained fractures at a median of 6.32 years (range 1.20–7.25 years) prior to the first DXA. The mean ± SD of spinal *Z*‐score, total hip *Z*‐score, spinal *T*‐score and total hip *T*‐score at first DXA for the fracture group were −2.13 ± 1.12, −2.15 ± 0.76, −2.16 ± 1.13, and −2.13 ± 0.71 respectively. The cumulative incidence at 5 and 9 years from the first DXA, with exception of the four having fractures prior to first DXA, were 4.11% (3/73) and 10.96% (8/73) respectively. The median age at first fracture was 31.73 years (range 24.06–44.18 years).

In the subgroup of participants who were naïve of bisphosphate treatment before the first DXA (*n* = 63), point prevalence of low BMD, bone fragility/osteoporosis and fracture were 65.1%, 7.9% and 12.7% respectively. In terms of spine, BMD *Z*‐score, 46.0%, 38.1% and 15.9% had *Z*‐score of above −2.0, between −2.0 and −3.0 and below −3.0 respectively. In terms of total hip, BMD *Z*‐score, 57.1%, 38.1% and 4.8% had *Z*‐score of above −2.0, between −2.0 and −3.0 and below −3.0 respectively. Among 27 patients who had never received bisphosphonate between the first and final DXA, eleven had low BMD *Z*‐score −2.0 or below. There was no statistically significant drop in hip or spine absolute BMD, BMD *Z*‐score or BMD *T*‐score between first and final DXA over 2.49 ± 1.02 years. For the percentage change in absolute BMD, there was a negative change (−1.2%) between first and final DXA in total hip BMD, but a positive change for spinal BMD (+3.5%) and hip neck BMD (+1.2%). Among this subgroup of patients, 7.4% (2/27) had sustained a fracture over 9 years.

When comparing the group having DXA data post‐bisphosphonate (*n* = 13) versus those having the DXA data before bisphosphonate treatment (*n* = 12), there are no statistically significant differences for spine *Z*‐score and total hip *Z*‐score (*p* > .05). Since only five participants had DXA scans both prior and post treatment of bisphosphonates, this makes meaningful statistical analysis on DXA data impossible. There was no statistical significant difference in terms of fracture occurrence between those received bisphosphonate versus those naïve of bisphosphonates (fracture occurred in 5/28 in bisphosphonate group versus 7/49 in bisphosphonate‐naïve group, *p* > .05). Two participants sustained fractures before any bisphosphonate treatment and was therefore treated as bisphosphonate‐naïve during the analysis. For those who continued to have fracture(s) post‐bisphosphonate, fracture(s) occurred at a median duration of 57 months (ranged from 14 to 124 months) from the last dose of bisphosphonates, which was not statistically associated with total duration of bisphosphonate treatment. Wide variety of dosage regimen and types of bisphosphonates received had made further comparison difficult.

The univariate analyses to assess for risk factors for fracture occurrence were shown in Table 2. In regression analysis, a higher log(10) transformation of average ferritin levels over 5 years before the first DXA (OR 310.73, 95% CI 3.99–24183.89, *p* = .010) and dosage of oral iron chelator deferasirox (Exjade^®^) in log(10) transformation (OR 1.99, 95% CI 1.13–3.52, *p* = .018) were significantly associated with fracture occurrence in positive manners, regardless of bisphosphonate treatment. The mean average ferritin level over 5 years was 6695.5 ± 2365.7 pmol/L in the fracture group which was significantly higher than 4350.7 ± 3103.2 pmol/L in the non‐fracture group (*p* = .016). However, there was no association between fracture occurrence and average annualized ferritin 1 or 2 year(s) before the first DXA. The average ferritin over 5 years was statistically higher in the deferasirox group (Ferritin level 6252.45 ± 2641.87 pmol/L in deferasirox group versus 4409.55 ± 3087.39 pmol/L in deferasirox‐naïve group, *p* < .05). There was a statistical significantly lower adjusted serum calcium level for the fracture group (*p* = .002). The proportion of patients with hypoparathyroidism was higher in the fracture group (16.7% in fracture group vs. 4.6% in non‐fracture group), although it failed to reach statistical significance. Vitamin D level was not routinely checked due to laboratory costing issue and thus resulted in missing data. There were no statistical significant associations between occurrence of fracture with age, sex, spinal or total hip BMD *Z*‐score, bisphosphonate treatment prior to fracture, gonadal dysfunction, hypoparathyroidism, proportion of patients receiving Vitamin D and calcium supplementation were demonstrated in the regression analysis. The proportion of patients on hormonal replacement therapy for gonadal failure also did not differ between the groups. The fracture group had mean spine and total hip height‐adjusted BMD *Z*‐score of below −2.0 compared with above −2.0 for non‐fracture group, although this difference did not reach statistical significance.

**TABLE 2:** Univariate analysis for fracture risk prediction

	Fracture (*n* = 12)	No fracture (*n* = 65)	*p*‐Value
Current age	39.12 ± 5.10	36.75 ± 9.29	.054
Age at 1st DXA	30.75 ± 5.49	28.24 ± 9.73	.070
Weight (kg)	49.95 ± 10.00	49.66 ± 8.76	.567
Height (cm)	151.68 ± 5.98	155.00 ± 9.24	.188
Gender			
Male	7	32	.759
Female	5	33	
HAZ spine BMD	−2.13 ± 1.12	−1.73 ± 0.96	
HAZ total hip BMD	−2.15 ± 0.76	−1.57 ± 1.19	
PC transfused (ml/kg/year)	241.7 ± 33.0	241.7 ± 52.8	.999
Liver iron overload, MRI T2*	9 (75.0%)	38 (65.5%)	.524
Average annualized ferritin over 5 year before 1st DXA scan (pmol/L)	6695.5 ± 2365.7	4350.7 ± 3103.2	.016
DFO (mg/day), mean ± SD	1000.00 ± 1066.00	1311.54 ± 980.31	.306
Deferiprone (mg/day), mean ± SD	2222.82 ± 2090.91	1761.97 ± 2675.78	.174
Deferasirox (mg/day), mean ± SD	737.50 ± 848.56	119.23 ± 378.52	.029
Chelator type			
SC	0 (0.0%)	8 (12.3%)	.437
Oral	5 (41.7%)	23 (35.4%)	
SC + oral	7 (58.3%)	34 (52.3%)	
No. of participant received bisphosphonates	5 (41.7%)	23 (35.4%)	.749
Bisphosphonate regimen	Pamidronate 30 mg/month (*n* = 3) 20 mg once (*n* = 1) Alendronate 10 mg/day (*n* = 1)	Pamidronate 30 mg/month (*n* = 7) 15 mg/month (*n* = 1) Alendronate 70 mg/week (*n* = 15) 10 mg/day (4) Ibandronic acid 150 mg Q3 month (*n* = 2) 2 agents received (*n* = 4) 3 agents received (*n* = 1)	N.A.
Bisphosphonate duration	27.17 ± 28.70 months	31.12 ± 28.44 months	.660
Calcium supplementation	9 (75.0%)	32 (47.7%)	.082
Adjusted calcium level (blood), mmol/L	2.15 ± 0.12	2.26 ± 0.11	.002
Vitamin D supplementation	7 (58.3%)	25 (38.5%)	.199
Gonadal dysfunction	9 (75.0%)	30 (46.2%)	.066
Percentage on HRT in those with gonadal dysfunction	88.9% (8 of 9)	83.3% (25 of 30)	1.000
Diabetes mellitus	4 (33.3%)	9 (13.8%)	.098
Thyroid dysfunction	6 (50.0%)	23 (35.4%)	.337
Obesity and overweight	3 (25.0%)	9 (13.8%)	.328
Hypoparathyroidism	2 (16.7%)	3 (4.6%)	.120

Abbreviations: BMD, bone mineral density; DFO, desferrioxamine; DXA, dual‐energy bone densitometry; HAZ, height adjusted *Z*‐score; SC, subcutaneous.

## 4 DISCUSSION

The fracture prevalence of TDT in Hong Kong is comparable with the North American data, 15.58% (our cohort) vs. 12.1% (in North America).^4^ Up to 55.84% of patients in our cohort, comparable with the North American cohort, have low bone mineral density whereas only 5.19% patients have osteoporosis/bone fragility state based on the clinical fracture and DXA data. After adopting the latest more stringent ISCD definition, our cohort has lower prevalence of osteoporosis/bone fragility as compared with historical cohorts^12^ which depended on BMD data alone to define the osteoporosis/bone fragility state. As fracture is the main patient‐oriented outcome to be addressed for prevention, a more stringent definition for osteoporosis could help in better identifying the at the risk group and focus on potential room for intervention.

Previous study had shown a ferritin level of >1800 microgram/L was associated with higher risk of multiple endocrinopathies in thalassemia major and intermedia.^13^ Our cohort has shown a higher average annualized ferritin, 6695.5 ± 2365.7 pmol/L (equivalent to 2979.5 ± 1052.7 microgram/L), over 5 years before the median age of around 29 years was associated with occurrence of fractures regardless of status of bisphosphonate treatment. In this study, we have successfully demonstrated that the impact of iron overload on bone health is an insidious process, as illustrated by the fact that the average ferritin level over 5 years instead of those over 1–2 years was shown to be associated with fracture occurrence. There was convincing evidence that iron overloading, a common problem of TDT, poses adverse effect on bone health, directly via threats on the growth of osteoblast in context of skeletal iron overload^14^ and indirectly through causing endocrinopathies associated with adverse bone health.^15^ The scene is further complicated by compliance problem to iron chelation and the previously reported adverse effect of chelators on bone health.^6^, ^15^ It remained controversial whether the newer oral chelator for instance, deferasirox, is helping or causing detrimental effect on bone health.^16^, ^17^ In our cohort, combination iron chelator therapy is commonly practised and made interpretation more difficult. In our study finding, use of deferasirox was a confounder for the association between fracture and high average annualized ferritin instead of a genuine risk factor for fracture. Therefore, the impact of different types of iron chelator on bone health could not be answered in current study.

We failed to demonstrate a statistically significant difference in the height‐adjusted BMD *Z*‐scores between the fracture and non‐fracture group, although mean *Z*‐score was below −2.0 in the fracture group vs. above −2.0 in the non‐fracture group. This could be due to the small number of patients with fracture(s) in our cohort. However, this emphasizes bone micro‐architecture, apart from areal BMD, should be monitored for better ascertainment of adverse bone health in patients with TDT,^18^ in especially for those with a mean BMD *Z*‐score of below −2.0. Among those who never received bisphosphonates between two DXA assessments, though limited in sample size, there was a negative percentage change in total hip BMD but not spinal BMD, this may suggest the hip being a more sensitive area to iron overloading. A well‐designed prospective study with regular assessment of associated endocrinopathies and assessment of bone mineral density in conjunction with bone micro‐architecture evaluation for instance, by peripheral quantitative computed tomography (QCT), can potentially predict fracture risk better in patients with TDT.

It is important to define the clinical parameters that alert us on who are at risk of fracture and to act before its occurrence. Summarizing our findings, we propose yearly DXA monitoring for patients of TDT who had an average annualized ferritin level ≥4400 pmol/L (around one SD below mean found in the fracture group) for more than 2 years and aged ≥19 years (based on the earliest age of fracture in our cohort being 24.06 years and a 5‐year fracture incidence of 4.11% after first BMD *Z*‐score of −2.0). Peripheral QCT should be considered as an adjunct, if available, to further assess bone micro‐architecture to decide if earlier use of anti‐osteoporotic agent to prevent fracture occurrence is necessary. In view of the lower mean adjusted serum calcium level in the fracture group, we suggest regular monitoring of calcium, vitamin D and PTH levels to ensure adequacy of calcium and vitamin D supplementation and timely treatment of hypoparathyroidism.

## AUTHOR CONTRIBUTIONS

**Samantha Lai‐Ka Lee:** Conceptualization (equal); data curation (equal); formal analysis (equal); investigation (equal); methodology (equal); project administration (equal); writing – original draft (equal); writing – review and editing (equal). **Chi Kong Li:** Conceptualization (equal); data curation (equal); methodology (equal); resources (equal); supervision (equal); writing – review and editing (equal). **Raymond Siu Ming Wong:** Data curation (equal); resources (equal). **Wing Kwan Leung:** Conceptualization (equal); data curation (equal); formal analysis (equal); investigation (equal); methodology (equal); project administration (equal); resources (equal); writing – review and editing (equal).

## CONFLICT OF INTEREST

The author has no conflict of interest in regard to any aspect of the current study.

## Data Availability

The data that support the findings of this study are available from the first author upon reasonable request.
